# Programmable, Multiplexed DNA Circuits Supporting Clinically Relevant, Electrochemical Antibody Detection

**DOI:** 10.1021/acssensors.1c00790

**Published:** 2021-06-15

**Authors:** Sara Bracaglia, Simona Ranallo, Kevin W. Plaxco, Francesco Ricci

**Affiliations:** † Department of Chemical Science and Technologies, University of Rome, Tor Vergata, 00133 Rome, Italy; ‡ Department of Chemistry and Biochemistry, University of California Santa Barbara, CA93106 Santa Barbara, California, United States

**Keywords:** DNA nanotechnology, electrochemical biosensors, antibody monitoring, DNA circuits, DNA sensors

## Abstract

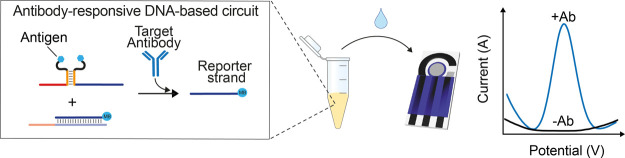

Current health emergencies have highlighted the need to have rapid, sensitive, and convenient platforms for the detection of specific antibodies. In response, we report here the design of an electrochemical DNA circuit that responds quantitatively to multiple specific antibodies. The approach employs synthetic antigen-conjugated nucleic acid strands that are rationally designed to induce a strand displacement reaction and release a redox reporter-modified strand upon the recognition of a specific target antibody. The approach is sensitive (low nanomolar detection limit), specific (no signal is observed in the presence of non-targeted antibodies), and selective (the platform can be employed in complex media, including 90% serum). The programmable nature of the strand displacement circuit makes it also versatile, and we demonstrate here the detection of five different antibodies, including three of which are clinically relevant. Using different redox reporters, we also show that the antibody-responsive circuit can be multiplexed and responds to different antibodies in the same solution without crosstalk.

The COVID19 pandemic has highlighted the crucial role that diagnostic tests can play in the detection, monitoring, and containment of infectious diseases.^1,2^ Different biomarkers can be used for such monitoring, but antibodies are among the most important as their detection not only reports on current and past infection but, in the latter case, also can inform on clinical outcomes.^3,4^ Antibody detection is likewise important in the treatment and monitoring of autoimmune diseases and cancer^5,6^ and, as antibodies are increasingly employed as therapeutic agents, in therapeutic drug monitoring.^7−10^

Recent years have seen extensive efforts to develop antibody detection strategies that are not only rapid, inexpensive, and easy to use but also quantitative, sensitive and useable at the point of care.^11,12^ Lateral flow immunoassays, thanks to their ease of use, their low cost, and their ability to work with unprocessed clinical samples, have become the uncontested leaders for antibody detection in point-of-care settings.^13^ Lateral flow assays, however, are usually qualitative, thus preventing their use in applications such as therapeutic monitoring, which requires precise quantitation.^14,15^ From this perspective, the ideal benchmark of an analytical point-of-care device remains without doubt the electrochemical glucose self-monitoring meter being not only quantitative but also cost-effective and easy to use.^16^ Electrochemical sensors are particularly well suited for point-of-care applications as they usually work well even when deployed directly in complex sample matrices, require low-cost instrumentation, can be mass-produced, and can be easily multiplexed.^17^

Recently, DNA nanotechnology, an emerging research field in which synthetic DNA strands are used to build structures and devices with nanoscale precision, has provided new sensing approaches for the detection of a wide range of targets.^18−23^ Among these methods, the design of DNA-based circuits in which different-responsive DNA synthetic strands react in a programmable way to give an output signal only in the presence of a specific target has given promising results.^24−27^ Several DNA-based circuits,^28,29^ for example, have been reported to date in which the detection of specific biomolecules has been achieved by optical- or colorimetric-based outputs.^30−33^ More recently, the electrochemical detection of specific genes and small molecules using DNA-based circuits has been also proposed.^34^

Motivated by the abovementioned considerations, we propose here the rational design of a DNA-based circuit that can be applied for the quantitative electrochemical detection of multiple, specific antibodies. The platform employs synthetic DNA strands as scaffolds for the conjugation of antibody-responsive elements and electrochemical signaling tags and allows us to couple the advantages of electrochemical detection with those of DNA-based circuits.^35^

## Results

**Figure 1:**
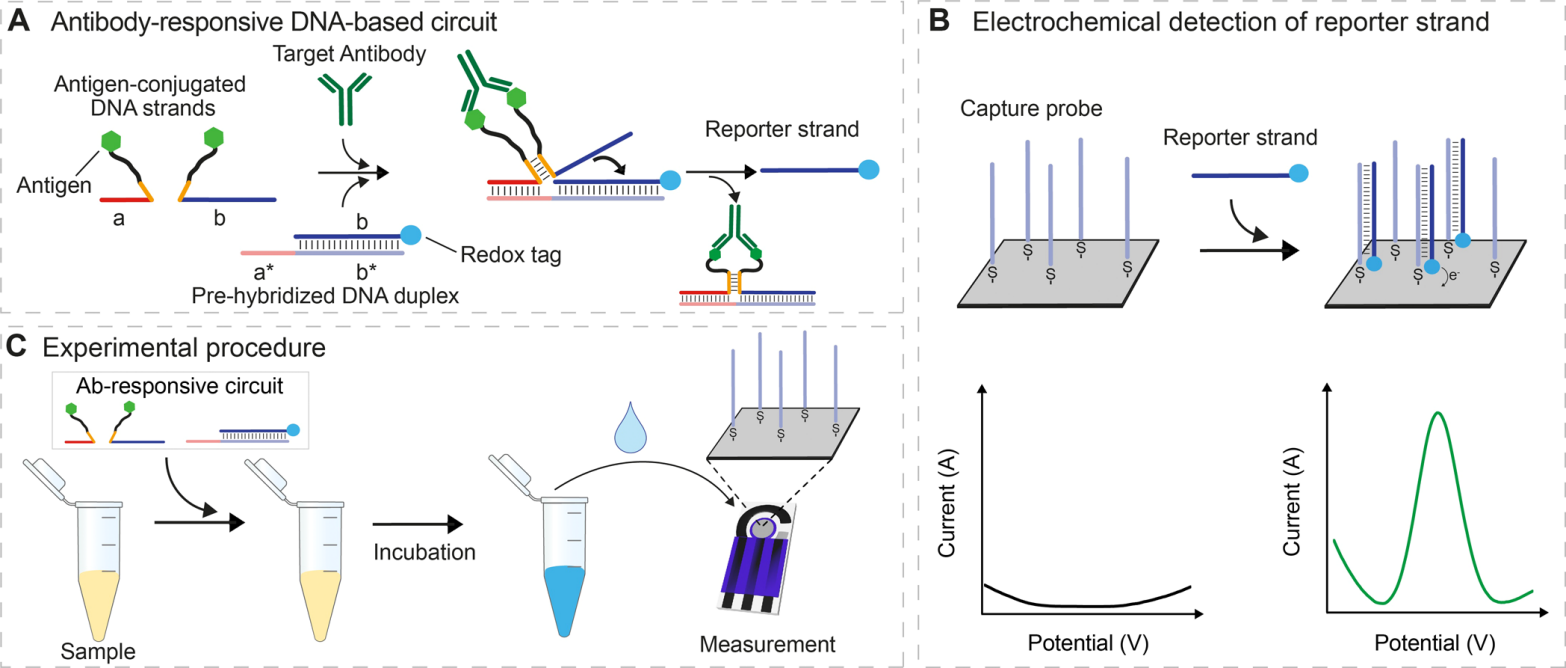
(A) Antibody-responsive nucleic acid circuit is made of a pre-hybridized duplex DNA containing the redox-labeled reporter and of two antigen-conjugated DNA strands. The binding of the target antibody to the two antigen-conjugated strands induces the formation of a functional complex able to activate a strand displacement reaction that releases the redox-labeled reporter strand from the pre-hybridized duplex. (B) Redox-labeled reporter strand can be detected through an electrochemical platform composed of a silver-based screen-printed disposable electrode on which a complementary DNA capture strand is immobilized. The hybridization of the reporter strand leads to a measurable electrochemical signal using square wave voltammetry (SWV). (C) Schematic of the measuring procedure: the antibody-responsive circuit elements are mixed with the sample of interest and incubated at room temperature (RT) and then transferred onto the screen-printed electrode surface for electrochemical measurement.

Our approach is based on the use of an antibody-responsive DNA strand displacement reaction (DNA “circuit”)^28,30^ re-engineered so that it can induce the release of a redox reporter-modified DNA strand in the presence of a specific target antibody. By combining such an antibody-responsive circuit with a disposable electrode on which a DNA capture sequence has been immobilized, we can achieve the sensitive and specific electrochemical quantitation of specific antibodies. The antibody-responsive circuit we have developed employs a set of three synthetic elements: a DNA duplex and two antigen-conjugated single-stranded DNAs. The duplex is composed of a 21-base redox reporter-modified strand and a 33-base strand that contains a 21-base fully complementary portion (denoted as “b*” in Figure 1A) but also includes an extra 12-base, single-stranded “toehold” domain (denoted as “a*” in Figure 1A). The two antigen-conjugated strands share a short complementary region (orange) connected to a 12-base poly-*T* linker (black) that terminates with a covalently attached antigen (Figure 1A). One of these two antigen-conjugated strands also includes a sequence (denoted as “a” in Figure 1A) complementary to the 12-base toehold of the pre-hybridized duplex. The other includes a sequence (denoted as “b” in Figure 1A) complementary to the 21-base strand in the duplex. Bivalent binding of the target antibody to the two antigen-conjugated DNA strands induces their co-localization, triggering in turn the hybridization of their short complementary regions, which would otherwise not form a duplex (orange, Figure 1A). The resulting complex binds to the toehold portion of the pre-hybridized duplex and invades it, releasing the redox reporter-modified single strand. This then hybridizes to the capture strand attached to the electrode, thus generating an easily measurable electrochemical signal (Figure 1B).

**Figure 2:**
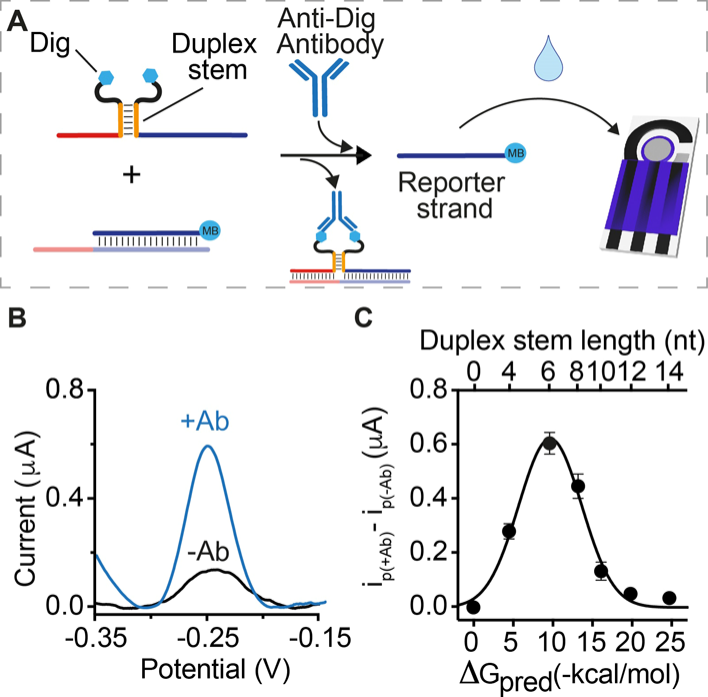
(A) Anti-Dig antibody-responsive circuit was used for signaling optimization. (B) SWV scans obtained in the absence (black) and presence (blue) of anti-Dig antibodies using Dig-conjugated DNA strands with 6-base complementary portions. (C) Plot showing the difference between the electrochemical signals obtained in the presence (*i*_p(+Ab)_) and absence (*i*_p(−Ab)_) of anti-Dig antibodies with Dig-conjugated DNA strands with variable lengths of the complementary portions. The experiments were performed in a 100 μL phosphate buffer solution (50 mM Na_2_HPO_4_, 150 mM NaCl, pH 7.0) containing the pre-hybridized DNA duplex (60 nM), Dig-conjugated DNA strands (100 nM each), and anti-Dig antibodies (300 nM). The antibody-responsive circuit was allowed to react for 30 min at RT after antibody addition and then transferred to the disposable electrode surface. SWV scans were performed between −0.35 and −0.15 V at 50 Hz.

Instrumental for the correct functioning of the circuit is the rational design of antigen-conjugated strands that only hybridize and thus form the complex required to release the reporter strand, upon being brought into proximity via antibody binding. As our design test bed, we first employed the small-molecule hapten digoxigenin (Dig) to target anti-Dig antibodies (Figure 2A). To do this, we designed a series of Dig-conjugated DNA strands differing in the length of their complementary regions, thus forming duplexes of varying stability (orange, Figure 2A). Specifically, we tested lengths ranging from 0 to 14 bases by recording SWVs in the absence and presence of saturating (300 nM) anti-Dig antibodies (Figure 2B). Doing so, we found that complementary regions longer than eight bases are not optimal because they are stable enough to produce measurable electrochemical signals even in the absence of anti-Dig antibodies (Figure S1). Shortening the complementary sequence, however, reduces this background until, at four bases, the background becomes undetectable (i.e., indistinguishable from that of a control construct lacking any complementarity). Bivalent binding of the targeted antibody to the two antigen-conjugated strands, on the other hand, produces measurable signals with complementary regions as short as four nucleotides (Figure S1). The greatest signal change between the absence and presence of the target antibody is achieved with six bases of complementarity between the two antigen-conjugated strands (Figure 2C), and thus, we employed this length in all following experiments.

**Figure 3:**
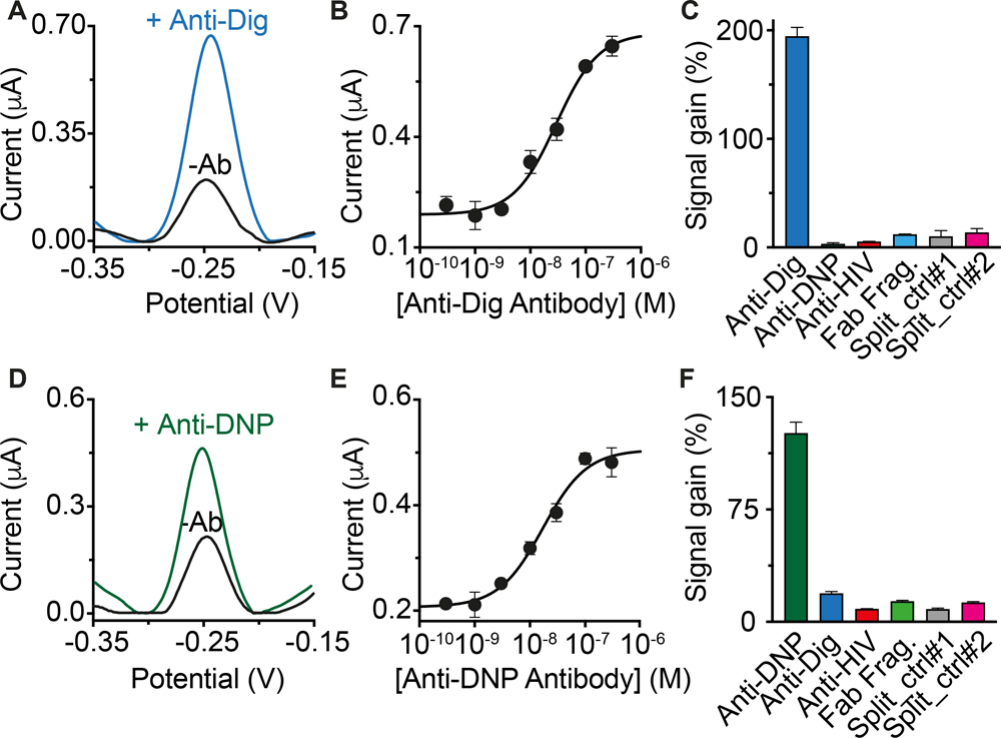
(A) SWV voltammograms obtained in the absence (black) and presence (blue) of anti-Dig antibodies using the optimized anti-Dig-responsive DNA circuit. (B) Current peak values obtained at increasing concentrations of anti-Dig antibodies. (C) Signal gain values observed at saturating concentration (300 nM) of anti-Dig antibodies, with non-specific antibodies and with different control experiments. Signal gain values are calculated as the relative signal change compared to the blank (−Ab) current signal. (D) SWV voltammograms, (E) dose–response curve, and (F) specificity tests obtained using a DNA-based circuit responsive to anti-DNP antibodies. The experiments were performed in a 100 μL solution containing 90% bovine serum and 10% buffer solution (500 mM Na_2_HPO_4_ and 1.5 M NaCl at pH 7.0). The solution also contains the pre-hybridized DNA duplex (60 nM), Dig/DNP-conjugated DNA strands (100 nM each), and anti-Dig/anti-DNP antibodies (300 nM). The antibody-responsive circuits were allowed to react for 30 min at RT and then transferred to the disposable electrode surface. SWV scans were performed between −0.35 and −0.15 V at 50 Hz after 120 min from the transfer of the solution on the electrode.

The optimized antibody-responsive circuit achieves the specific, sensitive, and convenient detection of anti-Dig antibodies in clinically relevant sample matrices. To see this, we challenged the anti-Dig-responsive circuit in antibody-doped, 90% bovine blood serum, a safe and convenient proxy for human samples (Figure 3A). In this matrix, the reaction kinetics is similar to that observed in buffer (Figure S2), and the limit of detection (defined as the concentration that reaches three standard deviations above a blank) is 9 ± 1 nM, above which we observe a concentration-dependent, approximately linear increase in the signal up to 130 nM (Figure 3B). We note here that such sensitivity, obtained without any amplification step, appears well suited for immunotherapy-monitoring applications where the expected serum concentration of therapeutic monoclonal antibodies reaches a high nanomolar range.^36^ Control experiments using other non-specific antibodies or an anti-Dig Fab fragment containing only a single binding site produce signals indistinguishable from those of target-free samples (Figure 3C). Control experiments employing an antigen-conjugated strand and a second strand lacking the antigen likewise support the antibody-induced co-localization mechanism proposed, as no measurable signal change is observed under these conditions (Figure 3D, Split_ctrl#1 and #2). Of note, the approach is quite convenient: in each of the abovementioned studies, the reaction between the antibody-responsive circuit and the sample was performed in a single Eppendorf tube for 30 min (longer times do not produce significantly higher signals, Figure S3) and then transferred to the disposable electrode surface for quantification (Figure 1C).

The antibody-detecting DNA circuit is generalizable to the detection of other antibodies via the simple expedient of changing the employed recognition element. To demonstrate this, we engineered an antibody-controlled circuit for the detection of anti-DNP antibodies. This circuit, also, detects its target with specificity and detection limits comparable to those we found for the detection of the anti-Dig (Figure 3D–F).

**Figure 4:**
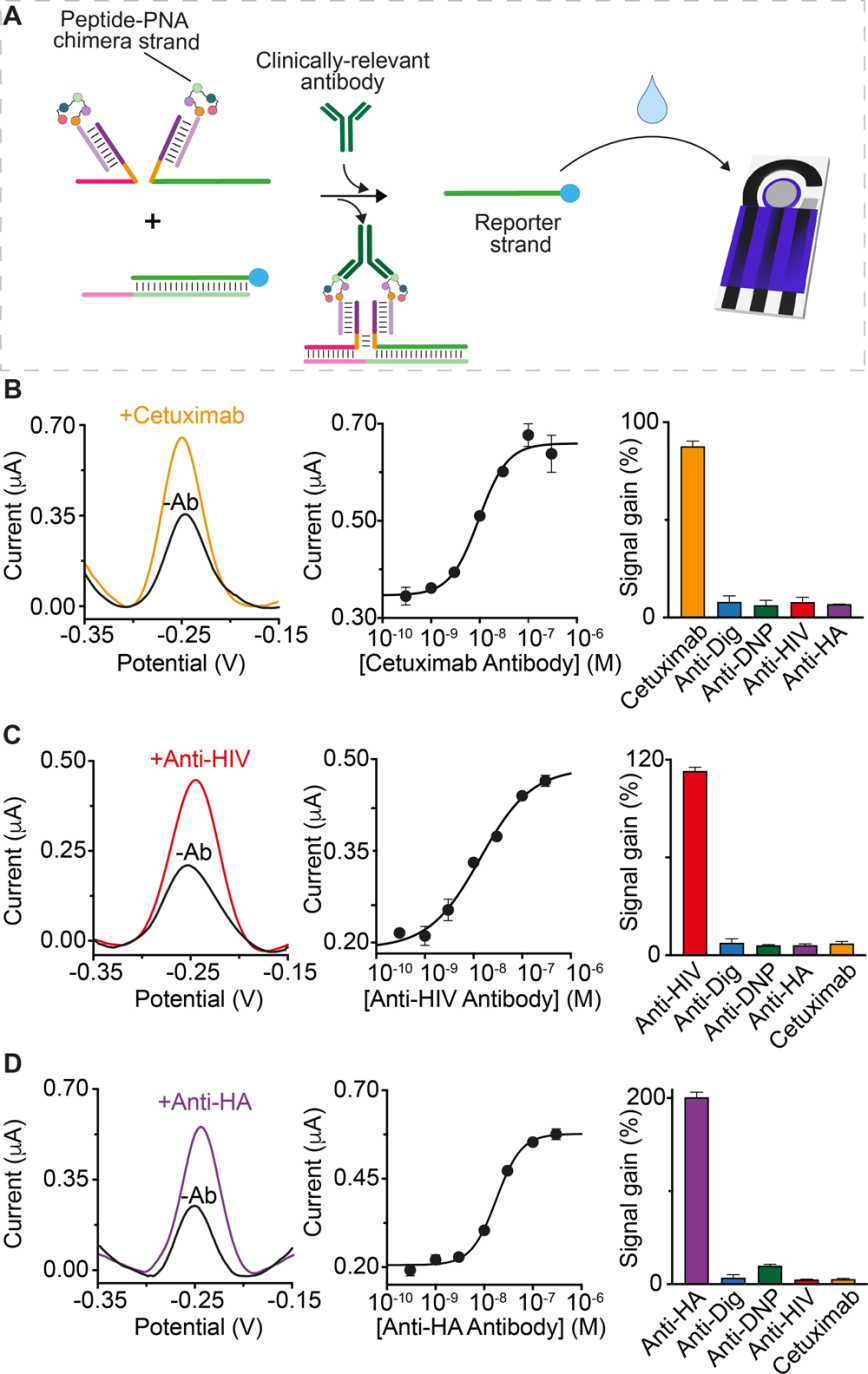
(A) Modular design of the electrochemical platform for the detection of three different clinically relevant antibodies: (B) cetuximab, (C) anti-HIV, and (D) anti-HA antibodies. Shown are (left) the SWV voltammograms recorded in the absence (blank) and presence (colored) of the target antibody, (center) the dose–response curves, and (right) the signal gain values obtained with the specificity tests. The experiments were performed in a 100 μL solution containing 90% bovine serum and 10% phosphate buffer (500 mM Na_2_HPO_4_ and 1.5 M NaCl at pH 7.0). Each solution also contains the pre-hybridized DNA duplex (60 nM), the scaffold DNA strands (60 nM each), and the peptide-PNA chimera (120 nM) and cetuximab/anti-HIV/anti-HA antibodies. Both the SWV examples and the specificity tests were performed at saturating concentrations of the target antibody (300 nM). The same experimental procedure described in Figure 3 was employed.

The majority of clinically relevant antibodies recognize proteins, and thus, we have also adapted the circuit to the detection of peptide epitope-recognizing antibodies. To reduce the cost of synthesizing the necessary antigen-modified DNA strands, we designed a modular version of the antibody-responsive circuit that allows the use of a single antigen-conjugated strand that hybridizes to two unmodified scaffold DNA strands (one of which contains a frame inversion), thus affording a more modular platform (Figure 4A). We also employed PNA, rather than DNA, as this is easier to conjugate a peptide to it. To demonstrate utility in the detection of peptide epitope-recognizing antibodies, we have characterized sensors displaying three clinically relevant peptide antigens: a 12-residue peptide excised from the epidermal growth factor receptor and recognized by cetuximab (a monoclonal antibody used as a therapeutic drug),^36^ a 13-residue epitope excised from the HIV protein p17 and recognized by anti-HIV antibodies,^37^ and a 9-residue epitope excised from the human influenza hemagglutinin (HA) protein and recognized by anti-HA antibodies.^38^ For all these electrochemical circuits, we achieved sensitivities and specificities comparable to those of the non-modular platforms (Figure 4B–D).

**Figure 5:**
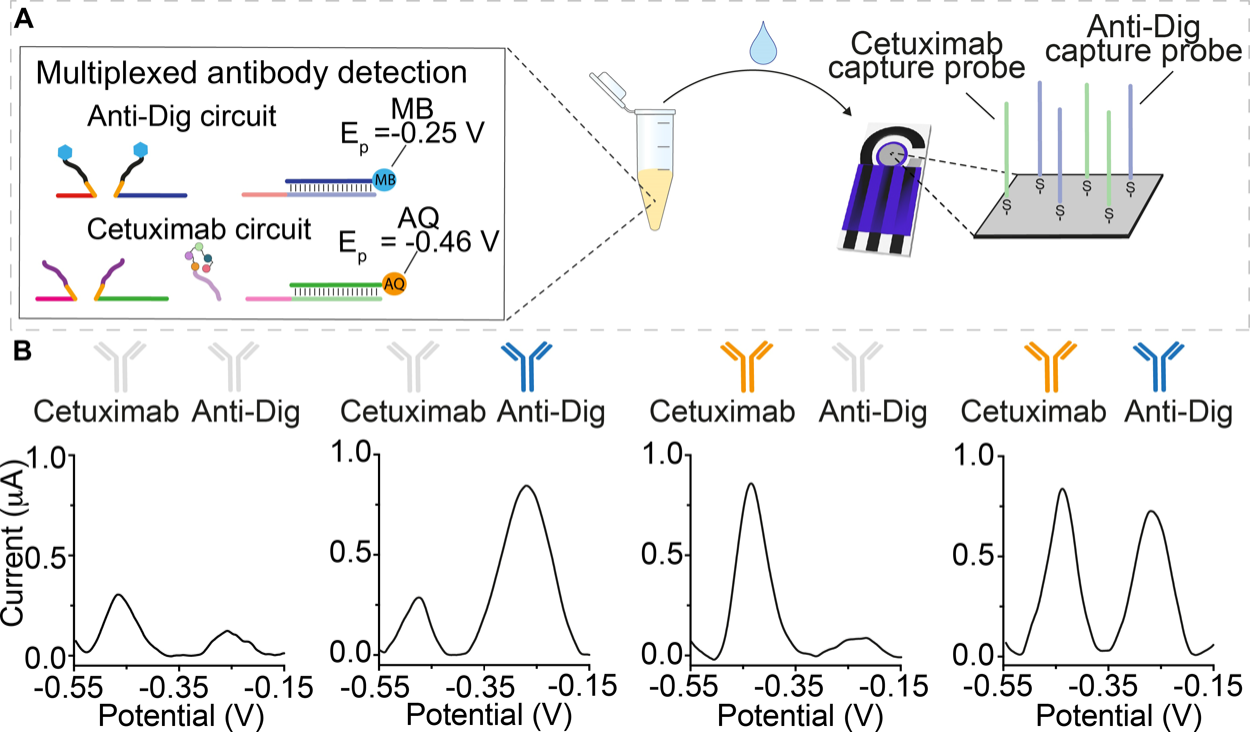
(A) Multiplex detection of anti-Dig (blue) and cetuximab (orange) antibodies using two orthogonal-responsive circuits releasing two different reporter strands labeled with two non-interfering redox labels (i.e., methylene blue, MB, and anthraquinone, AQ). (B) SWV profiles achieved for different experiments performed in the absence of both antibodies (left), in the presence of only one antibody (center), and in the presence of both antibodies (right). The colored antibody image identifies the added target for each experiment. The experiments were performed in a 100 μL phosphate buffer (50 mM Na_2_HPO_4_, 150 mM NaCl, pH 7.0) solution containing the two antibody-responsive circuits as previously described. The SWV voltammograms were obtained at saturating concentrations of the target antibody (300 nM).

The antibody-responsive DNA circuit also supports the simultaneous measurement of multiple antibodies in a single sample solution. To demonstrate this, we immobilized two distinct capture probes on one electrode. These were designed to hybridize the reporter strands of orthogonal antibody-responsive circuits for the detection of anti-Dig antibodies and cetuximab and modified with the redox reporters methylene blue and anthraquinone, respectively (Figure 5A). As the redox potentials of these reporters do not overlap, this allows the two strands to be monitored independently. The two circuits were mixed in the same Eppendorf tube and challenged with various combinations of their target antibodies. As expected, each responds to its specific antibody, generating a separated faradic current peak, and only in the presence of both antibodies, we achieved the two current peaks corresponding to the two circuits employed (Figure 5B).

## Conclusions

We have developed an electrochemical DNA circuit that responds quantitatively to multiple specific antibodies. The approach is sensitive (low nanomolar detection limit), specific (no signal is observed in the presence of non-targeted antibodies), and selective (the platform can be employed in complex media, including 90% serum). It is also versatile: this preliminary study has already demonstrated the detection of five different antibodies, including three of which are clinically important and are detectable at clinically relevant concentrations. The average serum levels of cetuximab during treatment, for example, are in the high nanomolar range.^36^ Finally, the antibody-responsive circuit is easily multiplexed: via the use of distinct redox reporters, circuits responding to different antibodies can be employed in the same solution without significant crosstalk.

The use of synthetic DNA oligonucleotides coupled with electrochemical detection affords potentially significant benefits for antibody detection compared to optical-based approaches. First, the platform is reagentless and convenient. The antibody-responsive circuit can be completed, for example, in 30 min in a single Eppendorf tube and then simply transferred to the surface of a disposable sensor. Second, compared to optical/colorimetric approaches,^39,40^ our electrochemical platform appears better suited for use in complex clinical sample matrices without any dilution or washing step and works well even in 90% serum. Finally, the portability and low cost of electrochemical instrumentation and the cost effectiveness of disposable electrodes render electrochemical approaches easily adaptable to point-of-care formats.^22^ Given these attributes, we believe that the electrochemical antibody-responsive circuits we have presented may prove to be well positioned for adaptation to point-of-care diagnostics.

## Materials and Methods

### Chemicals

Reagent-grade chemicals [sodium chloride (NaCl), magnesium chloride (MgCl_2_), disodium hydrogen phosphate (Na_2_HPO_4_), 6-mercapto1-hexanol (HS(CH_2_)_6_OH), Tris(2-carboxyethyl)phosphine (TCEP) (C_9_H_15_O_6_P)], fetal bovine serum, and mouse monoclonal anti-DNP antibodies were purchased from Sigma-Aldrich (St Louis, Missouri) and used without further purifications. Sheep polyclonal anti-Dig antibodies, the anti-Dig Fab fragment, and anti-HA antibodies were purchased from Roche Diagnostic Corporation (Germany), anti-DNP Fab fragments were purchased from Creative Biolabs, USA, murine monoclonal anti-HIV antibodies were purchased from Zeptometrix Corporation, and cetuximab antibodies were obtained from Merck (Darmstadt, Germany). All the antibodies were aliquoted and stored at 4 °C for immediate use or at 20 °C for long-term storage. Substrates used for printing electrodes (polyesters, Autostat HT5, *d* = 0.175 mm) were purchased from Autotype, Milan. Inks were delivered by Henkel (Milan) and were of different types: Elettrodag PF497A based on graphite; Electrodag PF410 based on silver; and Elettrodag 6018SS for the insulator.

### Preparation of DNA-Modified Electrodes

The DNA capture probe (100 μM) was reduced for 1 h in a solution of 0.4 mM Tris(2 carboxyethyl)phosphine hydrochloride (TCEP) prepared in 150 mM NaCl and 50 mM NaH_2_PO_4_, pH 7.0, to allow reduction of disulfide bonds. This solution was diluted to a final concentration of 100 nM in the same buffer. The DNA capture probe (20 μL) was dropcast only onto the silver working electrode of the SPE. After 1 h of incubation, SPE was rinsed with water, and 20 μL of 2 mM mercaptohexanol (prepared in 150 mM NaCl, 50 mM NaH_2_PO_4_, pH 7.0) was dropcast only onto the WE of the SPE to displace non-specifically adsorbed DNA and passivate the electrode area. After 1.5 h of incubation, SPE was rinsed with water.

### Oligonucleotides and DNA Circuits

HPLC-purified oligonucleotides were purchased from IBA (Gottingen, Germany) or Biosearch Technologies (Risskov, Denmark). All the DNA sequences used in this work are reported in the Supporting Information document.

### Electrochemical Experiments

All electrochemical measurements were performed at room temperature using an EmStatMUX potentiostat multiplexer (Palmsens Instruments, Netherland). The Ab-responsive circuits were allowed to react in an Eppendorf tube for 30 min at RT and then transferred to the disposable electrode surface. Experimental data were collected using square wave voltammetry from −0.15 to −0.35 V in increments of 0.001 V versus Ag/AgCl, with an amplitude of 10 mV and a frequency of 50 Hz for when using methylene blue as a redox label. Square wave voltammetry for the circuit that employs anthraquinone was performed from −0.1 to −0.55 V in increments of 1 mV versus Ag/AgCl, with an amplitude of 50 mV and a frequency of 50 Hz. Peak currents were fit using the manual fit mode in PSTrace 4.5v software (of Palmsens Instrument). All experiments were performed in a 100 μL phosphate 50 mM Na_2_HPO_4_, 150 mM NaCl, pH 7.0 solution at 25 °C.

### Data Analysis

Binding curves were fit with the following four-parameter logistic equation
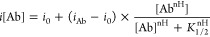
where *i*[Ab] is the current observed in the presence of a given concentration of the target analyte; i_0_ is the background current observed in the absence of the target analyte; [Ab] is the antibody concentration; *i*_(Ab)_ is the current seen in the presence of saturating concentration of the target; *K*_1/2_ is the concentration at half of the maximum signal change; and nH is the Hill coefficient. Signal gain (%) values used to compare specificity experiments are calculated as the relative signal change registered upon the addition of saturating concentration of target antibodies (or non-specific antibodies) using the following formula
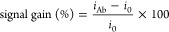
where *i*_(Ab)_ = current in the presence of different concentrations of the antibody; *i*_0_ = background current. The limit of detection (LOD) was determined as the concentration that reaches three standard deviations above a blank.

## Supporting Information Available

DNA circuit sequences and electrochemical kinetic traces of the anti-Dig-responsive circuit (PDF)
