# Supplementary material for: Programmable, Multiplexed DNA Circuits Supporting Clinically Relevant, Electrochemical Antibody Detection
Source: ACS Sens. 2021 Jun 15;6(6):2442–8. doi: 10.1021/acssensors.1c00790 (PMC8240086; doi:10.1021/acssensors.1c00790)
Supplement: Supplementary file 1 — se1c00790_si_001.pdf [file se1c00790_si_001.pdf]

Supplementary Information:

Programmable, Multiplexed DNA Circuits Supporting Clinically Relevant, Electrochemical Antibody Detection

Sara Bracaglia,[1] Simona Ranallo,[1,2,]* Kevin W. Plaxco[2] and Francesco Ricci[1,] *

[1]Department of Chemical Science and Technologies, University of Rome Tor Vergata, 00133, Italy;

[2]Department of Chemistry and Biochemistry, University of California Santa Barbara, CA93106, United States;

Supplementary Methods

DNA Sequences

HPLC purified oligonucleotides were purchased from IBA (Gottingen, Germany), Biosearch Technologies (Risskov, Denmark) and Metabion International AG (Planegg, Germany). PNA/Peptide chimera probes were purchased from Panagene (South Korea). All sequences were designed using Nupack or IDT oligoanalyzer tools.[1,2]

Table S-1: Anti-Dig responsive circuit

**Name**	**Sequence (5' - 3')**
S1	CCT CAT CAT CAT ATA CGT CAC **CTA TCC CAT TCT**
Capture probe	(Thiol C6 SS) - CCT CAT CAT CAT ATA CGT CAC
Reporter strand	GTG ACG TAT ATG ATG ATG AGG - (Methylene Blue)
Split#1_Stem6	**AGA ATG GGA TAG** – TT – *GTC TGC* – TTT TTT TTT TTT - (DIG)
Split#2_Stem6	(DIG) - TTT TTT TTT TTT – *GCA GAC* – TT – GTG ACG TAT ATG ATG ATG AGG
Split#1_Stem4	**AGA ATG GGA TAG** - TT - *G TCT* - TTT TTT TTT TTT - (DIG)
Split#1_Stem8	**AGA ATG GGA TAG** – TT – *G TCT GCT G* – TT TTT TTT - (DIG)
Split#1_Stem10	**AGA ATG GGA TAG** – TT – *G TCT GCT GTC* – TTT TTT TT - (DIG)
Split#1_Stem12	**AGA ATG GGA TAG** – TT – *C ATC GTC GTC TGC TG* – TT TTT TTT TTT - (DIG)
Split#1_Stem14	**AGA ATG GGA TAG** – TT – *CAT CGT CTG CTG TC* – TT TTT TTT TTT T - (DIG)
Split#2_Stem0	(DIG) - TTT TTT TTT TTT – *TGG GTG* – TT – GTG ACG TAT ATG ATG ATG AGG
Split#2_Stem4	(DIG) - TTT TTT TTT TTT – *AGA C* – TT – GTG ACG TAT ATG ATG ATG AGG
Split#2_Stem8	(DIG) - TTT TTT TTT TT – *C AGC AGA C* – TT – GTG ACG TAT ATG ATG ATG AGG
Split#2_Stem10	(DIG) - TTT TTT TT – *G ACA GCA GAC* – TT – GTG ACG TAT ATG ATG ATG AGG

Split#2_Stem12	(DIG) - TTT TTT TTT TT – *C AGC AGA CGA TG* – TT – G TGA CGT ATA TGA TGA TGA GG
Split#2_Stem14	(DIG) - TTT TTT TTT TT – *G ACA GCA GAC GAT G* – TT - GTG ACG TAT ATG ATG ATG AGG
Split_Ctrl#1	**AGA ATG GGA TAG** – TT – *GTC TGC* – TTT TTT TTT TTT
Split_Ctrl#2	TTT TTT TTT TTT – *GCA GAC* – TT – GTG ACG TAT ATG ATG ATG AGG

In the above sequences and those reported below with other circuits the sequence denoted in **bold** indicates the toehold domain, the underlined sequence represents the invading domain, and the sequence denoted in *italics* indicates the elements of the duplex stem.

In the above antigen-conjugated DNA strands Dig was conjugated using EDC/NHS to an amine attached via a 5-carbon linker on either the 5'- or 3'-end of the DNA. The methylene blue (MB) redox reporter for this and other DNA strands was conjugated to the 3′- amino modification on the relevant DNA using succinimide ester coupling

Table S-2: Anti-DNP responsive circuit

**Name**	**Sequence (5' - 3')**
S1	CCA ATA TCA TCA AGA GCG TAC – **GAT TCG TGA GTA**
Capture Probe	(Thiol C6 SS) - CCA ATA TCA TCA AGA GCG TAC
Reporter strand	GTA CGC TCT TGA TGA TAT TGG - (Methylene Blue)
Reporter strand_AQ	GTA CGC TCT TGA TGA TAT TGG - (Anthraquinone)
Split#1	**TAC TCA CGA ATC** - TT - *G TCT GC* - T TTT TTT TTT TT - (DNP)
Split#2	(DNP) - TTT TTT TTT TTT - *GCA GAC* - TT - G TAC GCT CTT GAT GAT ATT GG
Split_Ctrl#1	**TAC TCA CGA ATC** - TT - *G TCT GC* - T TTT TTT TTT TT
Split_Ctrl#2	TTT TTT TTT TTT - *GCA GAC* - TT - G TAC GCT CTT GAT GAT ATT GG

In these antigen-conjugated strands DNP was attached via a triethylene glycol (TEG) spacer arm on either the 5'- or the 3'- terminus of the DNA. The anthraquinone (AQ) redox reporter was conjugated to the 3′-end of the relevant DNA strand through a phosphoramidite chemistry.

Modular antibody-responsive circuit:

We have also designed a version of the antibody-responsive circuit that, by employing a a frame inversion in one of the scaffold strands, does not require the synthesis of two different antigen-conjugated strands (one in each orientation). Instead, this employs a single antigen-conjugated strand that hybridizes to both scaffold strands. We employed this with the peptide antigens in our study (as their synthesis is the most expensive). For these, we also employed a PNA, rather than a DNA, antigen-conjugated strand so as to simplify the conjugation chemistry.

Table S-3: Clinical antibody responsive circuit

Name	Sequence
Split#1_Scaffold	5' - **TAC TCA CGA ATC** - TT - *G CCT GA* - C CCT - AGA ATA AAA CGC CAC TG - 3'
Split#2_Scaffold	3' - GTC ACC GCA AAA TAA GA - 5' 5'- TCC C - *TC AGG C* - TT - <u>GTA CGC TCT TGA TGA TAT TGG</u> - 3'
P17_PNA chimera	N_{term} -(ELDRWEKIRLRP) - CAG TGG CGT TTT ATT CT - C_{term}
epitope_PNA chimera	N_{term} - (CVFDLGTRRLRCGGG) - CAGTGGCGTTTTATTCT-C_{term}
HA_PNA chimera	N_{term} – (YPYDVPDYA) - CAG TGG CGT TTT ATT CT - C_{term}

The sequence in parentheses represent the selected epitope portions that are terminally conjugated to PNA. The sequences of the peptide epitopes are set in parentheses.

Supplementary Figure

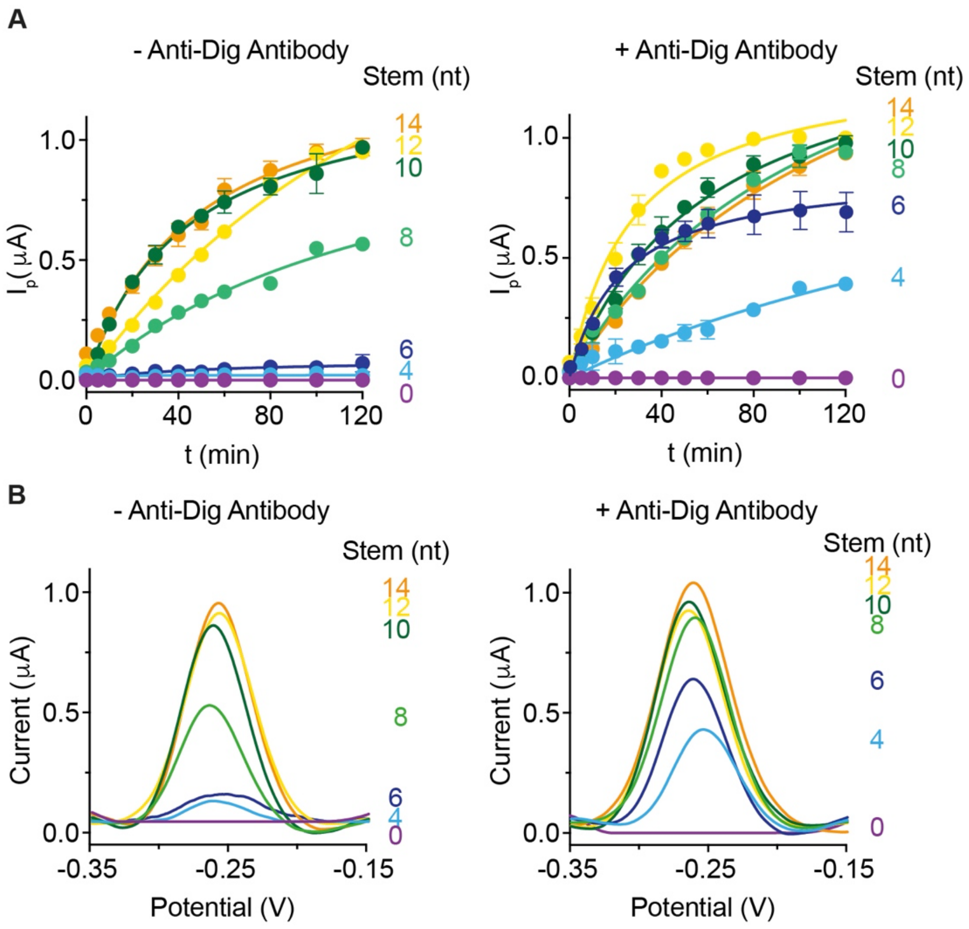

Figure S1: Electrochemical kinetic traces obtained by recording SWV of the anti-Dig responsive circuit for a series of Dig-conjugated DNA strands differing in the length of their complementary (from 0 to 14 bases) in the absence (A, left) and presence (A, right) of saturating anti-Dig antibody concentration. The Ab-responsive circuit was allowed to react for 30 min at RT after antibody addition and then transferred to the disposable electrode surface. (B) SWV scans were performed between -0.35 and -0.15 V at 50 Hz, after 120 min from the transfer of the solution on the electrode. The experiments were performed in a 100 μL phosphate buffer solution (50 mM Na_2HPO_4, 150 mM NaCl, pH 7.0) containing the pre-hybridized DNA duplex (60 nM), Dig-conjugated DNA strands (100 nM each) and Anti-Dig antibodies (300 nM).

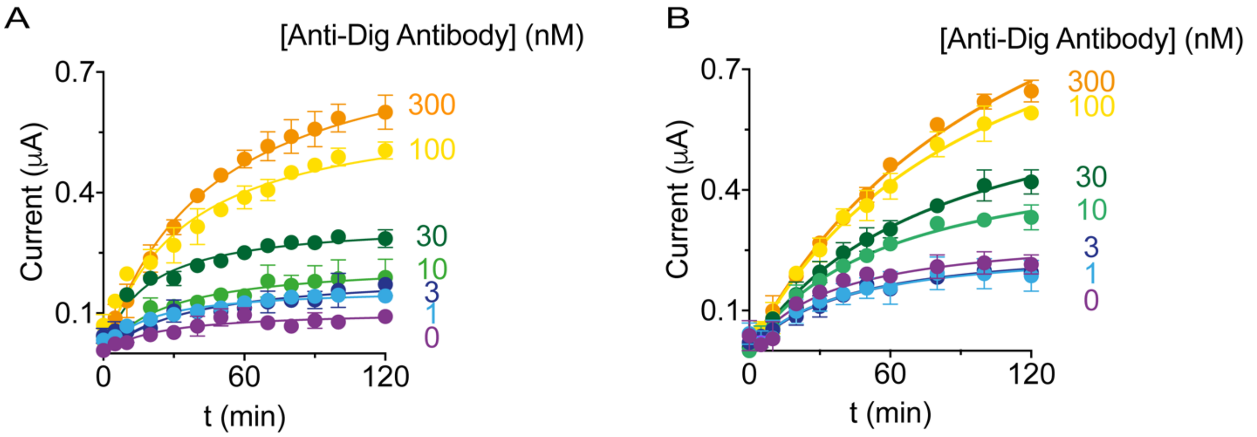

Figure S2: Electrochemical kinetic traces obtained by recording SWV at increasing concentration of Anti-Dig antibody preformed (A) in pure buffer (50 mM Na_2HPO_4, 150 mM NaCl, pH 7.0) and (B) 90% serum diluted with 10% of 10X buffer (500 mM Na_2HPO_4, 1.5 M NaCl, pH 7.0). The experiments were performed in a 100 μL drop solution containing the pre-hybridized DNA duplex (60 nM), Dig-conjugated DNA strands (100 nM each) and Anti-Dig antibodies (300 nM).

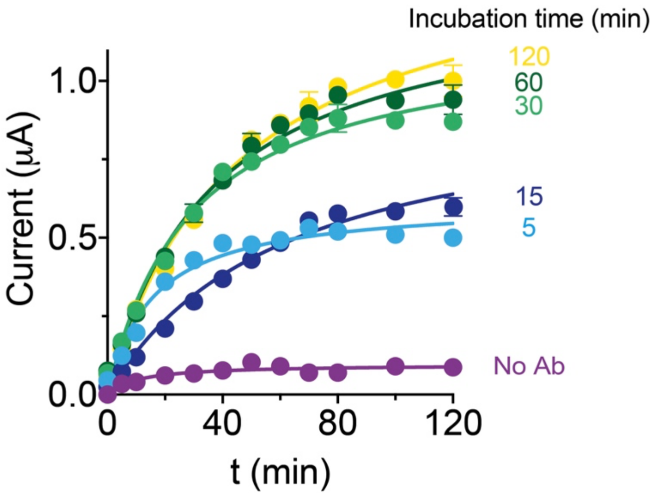

Figure S3: Kinetics of target hybridization at different incubating times after addition of a saturating concentration of Anti-Dig antibody. The experiment was performed in a 100 μL phosphate buffer solution (50 mM Na_2HPO_4, 150 mM NaCl, pH 7.0) containing the pre-hybridized DNA duplex (60 nM), Dig-conjugated DNA strands (100 nM each) and Anti-Dig antibodies (300 nM).

Supplementary References

(1) Zadeh, J. N.; Steenberg, C. D.; Bois, J. S.; Wolfe, B. R.; Pierce, M. B.; Khan, A. R.; Dirks, R. M.; Pierce, N. A. NUPACK: Analysis and Design of Nucleic Acid Systems. *J. Comput. Chem.* **2011**, *32* (1), 170–173.

(2) Owczarzy, R.; Tataurov, A. V.; Wu, Y.; Manthey, J. A.; McQuisten, K. A.; Almabrazi, H. G.; Pedersen, K. F.; Lin, Y.; Garretson, J.; McEntaggart, N. O.; Sailor, C. A.; Dawson, R. B.; Peek, A. S. IDT SciTools: A Suite for Analysis and Design of Nucleic Acid Oligomers. *Nucleic Acids Res* **2008**, *36* (Web Server issue), W163-169.
